# Mutational Spectrum, Ocular and Olfactory Phenotypes of *CNGB1*-Related RP-Olfactory Dysfunction Syndrome in a Multiethnic Cohort

**DOI:** 10.3390/genes14040830

**Published:** 2023-03-30

**Authors:** Sara Geada, Francisco Teixeira-Marques, Bruno Teixeira, Ana Luísa Carvalho, Nuno Lousan, Jorge Saraiva, Joaquim Murta, Rufino Silva, Xavier Zanlonghi, Sabine Defoort-Dhellemmes, Vasily Smirnov, Claire-Marie Dhaenens, Catherine Blanchet, Isabelle Meunier, João Pedro Marques

**Affiliations:** 1 Ophthalmology Unit, Centro Hospitalar e Universitário de Coimbra (CHUC), 3000-075 Coimbra, Portugal; 2 Department of Otorhinolaryngology, Centro Hospitalar do Tâmega e Sousa (CHTS), 4560-162 Penafiel, Portugal; 3 Medical Genetics Unit, Centro Hospitalar e Universitário de Coimbra, 3000-602 Coimbra, Portugal; 4 Clinical Academic Center of Coimbra (CACC), 3000-354 Coimbra, Portugal; 5 University Clinic of Medical Genetics, Faculty of Medicine, University of Coimbra (FMUC), 3000-354 Coimbra, Portugal; 6 University Clinic of Pediatrics, Faculty of Medicine, University of Coimbra (FMUC), 3000-354 Coimbra, Portugal; 7 University Clinic of Ophthalmology, Faculty of Medicine, University of Coimbra (FMUC), 3000-354 Coimbra, Portugal; 8 Eye Department, Rennes University Hospital, 35 033 Rennes, France; 9 Department of Visual Exploration and Neuro-Ophthalmology, Robert Salengro Hospital, 59 037 Lille, France; 10 Sensgene Care Network, 67 091 Strasbourg, France; 11 University of Lille, INSERM, CHU Lille, U1172-LilNCog-Lille Neuroscience & Cognition, 59 000 Lille, France; 12 Reference Centre for Inherited Sensory Diseases, Montpellier University Hospital, 34 295 Montpellier, France; 13 Institute for Neurosciences of Montpellier (INM), University of Montpellier, INSERM, 34 091 Montpellier, France

**Keywords:** inherited retinal disease, rod-cone degeneration, retinitis pigmentosa, olfactory dysfunction, *CNGB1*

## Abstract

*CNGB1* gene mutations are a well-known cause of autosomal recessive retinitis pigmentosa (RP), which was recently associated with olfactory dysfunction. The purpose of this study was to report the molecular spectrum and the ocular and olfactory phenotypes of a multiethnic cohort with *CNGB1*-associated RP. A cross-sectional case series was conducted at two ophthalmic genetics referral centers. Consecutive patients with molecularly confirmed *CNGB1*-related RP were included. All patients underwent a complete ophthalmological examination complemented by psychophysical olfactory evaluation. Fifteen patients (10 families: 8 Portuguese, 1 French, and 1 Turkish), mean aged 57.13 ± 15.37 years old (yo), were enrolled. Seven disease-causing variants were identified, two of which are reported for the first time: c.2565_2566del and c.2285G > T. Although 11/15 patients reported onset of nyctalopia before age 10, diagnosis was only established after 30 yo in 9/15. Despite widespread retinal degeneration being present in 14/15 probands, a relatively preserved visual acuity was observed throughout follow-up. Olfactory function was preserved in only 4/15 patients, all of whom carried at least one missense variant. Our study supports previous reports of an autosomal recessive RP-olfactory dysfunction syndrome in association with certain disease-causing variants in the *CNGB1* gene and expands the mutational spectrum of *CNGB1*-related disease by reporting two novel variants.

## 1. Introduction

Retinitis pigmentosa (RP) is the most frequent inherited retinal degeneration (IRD) [1], with an estimated prevalence of 1:4000 [1,2], and a major cause of visual impairment and blindness worldwide [2]. Usually, the first visual symptoms are night blindness and visual field constriction (primary rod dysfunction), with secondary cone involvement leading to decreased daylight and central vision later in the disease course [1,3]. Despite its progressive character, RP has great phenotypic variability, which is in line with its well-known genetic heterogeneity [1,3]. To date, genetic variants in at least 89 genes have been reported to cause RP [4], making it one of the most genetically heterogeneous diseases in humans [5]. While in most cases the condition is limited to the eye, extra-ocular manifestations (syndromic RP) are observed in 20–30% of cases [1,2], due to a significant expression of the involved gene transcript in other organs [6].

Photoreceptors and olfactory sensory neurons (OSNs) depend on cyclic nucleotide-gated (CNG) channels for signal transduction [6]. CNG channels are formed from different subunits, of which B1, encoded by the *CNGB1* gene, is the only subunit that is present in both rod photoreceptors and OSNs [6,7,8,9]. However, the complex structure of the *CNGB1* gene results in two different transcripts (CNGB1a and CNGB1b proteins) depending on the tissues where it is expressed. The full-length CNGB1a protein is exclusively expressed in the retina, predominantly in rods, and includes a long cytosolic N-terminus (glutamic acid-rich protein [GARP]) domain and a channel domain. The shorter transcript (CNGB1b protein) lacks the GARP domain and is expressed in OSNs [6,10,11,12,13]. Therefore, an abnormal CNG channel resulting from a mutation in *CNGB1* affecting at least the channel transcript would be expected to result in a compromised retinal rod phototransduction and lack of conversion of odor stimuli into electric signals by the OSNs, leading to both retinal degeneration and olfactory dysfunction [6,13,14,15]—an association that was established in mice by Hüttl et al. and Michalakis et al. [16,17].

Clinically significant *CNGB1* variants are a known cause of autosomal recessive RP (MIM#613767), characterized by the onset of night blindness during childhood but a relatively preserved visual acuity until the late stages of the disease [13,18]. On fundus examination, the condition is characterized by classic RP signs (bone spicule pigment clumping in the mid-peripheral and peripheral retina, vascular attenuation, and optic disc pallor). A parafoveal ring of hyperautofluorescence is usually present in fundus autofluorescence [6,13,18].

Even though *CNGB1*-related retinal degeneration is well described, only three studies have associated *CNGB1* variants with both RP and olfactory dysfunction [6,14,15]. The aim of this study was to report the molecular spectrum along with the ocular and olfactory phenotypes of a multiethnic cohort with genetically confirmed *CNGB1*-associated retinal degeneration.

## 2. Materials and Methods

### 2.1. Study Design and Patient Selection

We conducted a cross-sectional case series at two IRD reference centers and full members of the European Reference Network for Rare Eye Diseases (ERN-EYE): Centro Hospitalar e Universitário de Coimbra (CHUC), Coimbra, Portugal, and *Hôpital Gui de Chauliac*—Centre Hospitalier Universitaire (CHRU) de Montpellier, Montpellier, France. Patients with a clinical and molecular diagnosis of *CNGB1*-associated retinal degeneration were identified using the IRD-PT registry [19] in Portugal and the MAOLYA Registry in France and invited to participate. Clinical diagnosis was based on patient history, clinical examination, multimodal retinal imaging, and functional testing. The molecular diagnosis was established based on the identification of biallelic pathogenic (class V) or likely pathogenic (class IV) variants in the *CNGB1* gene in accordance with the American College of Medical Genetics and Genomics (ACMG) [20], using next-generation sequencing (NGS) methods as reported previously [21,22]. Probands from the Portuguese cohort underwent a whole exome sequencing (WES)-based NGS panel (302 IRD-associated genes—Supplemental Table S1) with copy number variations (CNV) screening. Probands from the French cohort underwent an NGS panel (238 IRD-associated genes—Supplemental Table S1). Whenever possible, segregation analysis using Sanger sequencing was conducted in both affected and unaffected family members. Genetic counseling provided by a medical geneticist was granted to all subjects. The study was approved by the local ethics committees and followed the tenets of the Declaration of Helsinki for biomedical research. Written informed consent was obtained for every included subject.

### 2.2. Classification of CNGB1 Variants

Identified variants were classified according to the ACMG classification [20], as mentioned above. Characteristics such as molecular consequence (nonsense, frameshift, splice site, missense, and in-frame mutations) and affected protein domain (GARP domain vs. channel domain) were highlighted.

### 2.3. Clinical/Demographic Features

Baseline demographics (age, gender, country of origin), symptoms, age at onset, family history, history of consanguinity, age at diagnosis, best-corrected visual acuity (BCVA, ETDRS (Early Treatment of Diabetic Retinopathy Study) letters) at baseline and throughout follow-up, and follow-up time were collected from each patient’s medical records.

The age at onset was defined as the age at which the first ocular symptoms were noted by the patient or his caregivers (in the case of childhood onset), but ascribing to the possible effect of recall bias, this feature was categorized into a timeframe: childhood (6–10 years); adolescence (11–20 years); early adulthood (21–30 years); adulthood (31–50 years); and elderly (>51 years).

Disease duration was calculated as the difference between the subject’s age at the cross-sectional visit and the age of onset.

### 2.4. Ophthalmic Examination and Multimodal Imaging

At the cross-sectional visit, all patients underwent a comprehensive ophthalmologic examination, including: (1) best-corrected visual acuity (BCVA), converted to equivalent ETDRS letters; (2) dilated slit-lamp anterior segment and fundus biomicroscopy; (3) multimodal imaging comprising color fundus photography (CFP) and blue-light fundus autofluorescence (FAF) imaging using, whenever possible, a widefield scanning laser ophthalmoscope; and spectral domain optical coherence tomography (SD-OCT).

### 2.5. Olfactory Function Evaluation

**Figure 1:**
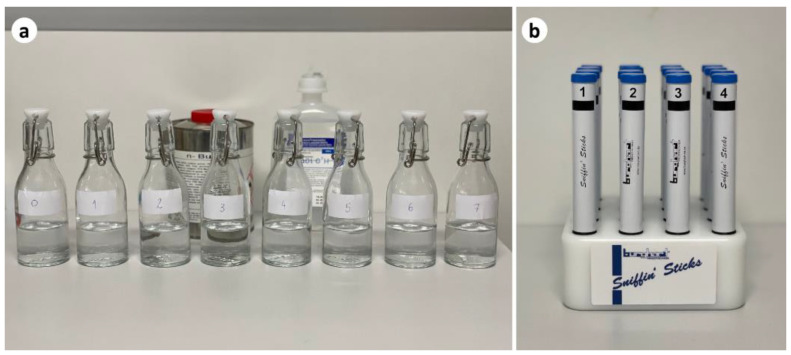
(**a**) The eight bottles used for the Connecticut Chemosensory Clinical Research Center odor threshold test: one blank (0) with deionized water and seven bottles with different dilutions of butanol (numbered 1 to 7); (**b**) the Sniffin’ Sticks^®^ 16 odor identification test with 16 pens.

A thorough medical history was taken in all patients, followed by a full otorhinolaryngology examination. Olfactory testing started with a subjective assessment with a visual analogue scale (VAS), where the score varied between 0 (absence of smell) and 10 (normal sense of smell). This was followed by psychophysical tests for odor threshold and identification, as recommended by Hummel et al. [23]. For odor threshold, we utilized the Connecticut Chemosensory Clinical Research Center (CCCRC) threshold test with butanol [24] (Figure 1a); and for odor identification, we used the Sniffin’ Sticks^®^ (SnSt) identification test with 16 pens (Burghart Messtechnik) (Figure 1b), which has been validated for the Portuguese language and is used in most European countries, including France [25,26,27].

### 2.6. Statistical Analysis

The study population’s demographics, clinical, and imaging characteristics were summarized by traditional descriptive methods using Microsoft Excel 365^®^. Statistical analysis regarding visual acuity variation during follow-up was performed using IBM SPSS Statistics 25 for Windows^®^. *p* < 0.05 was considered statistically significant.

## 3. Results

Demographic, clinical, and genetic data for every subject are represented in Table 1. A total of 15 patients from 10 families (8 of Portuguese origin, 1 of French origin, and 1 of Turkish origin) with a mean age of 57.13 ± 15.37 yo and a median disease duration of 38.00 ± 16.60 (range 10–78) years were enrolled in the study. The majority (13/15) presented a positive family history, but only 4 reported a history of consanguinity in the family.

Pedigrees are represented in Supplemental Figure S1.

### 3.1. CNGB1 Variants

Among the 10 families, 7 different clinically significant variants in the *CNGB1* gene (NM_001297.4) were identified (Table 1): 4 variants in the Portuguese families (two of which are novel); 1 variant in the French family; and 2 variants in the Turkish family. The c.1958-1G > A, p.? class V variant demonstrated a high allele frequency in the Portuguese families (7/8 families). It was observed in homozygosity in 4 families and in trans with 2 novel variants in 2 families: c.2565_2566del, p.(Phe856*) (class IV) and c.2285G > T, p.(Arg762Leu) (class IV). The latter, along with the single variant observed in the French family, c.2978G > T, p.Gly993Val (Class IV), were the only missense mutations found. Regarding the Turkish family, two different variants were observed: c.2492 + 2T > G, p.? (Class V), present in homozygosity in patient 14J and in compound heterozygosity along with the variant c.1917G > A, p.(Trp639*) (Class V) in patient 15J (14J’s nephew).

All identified mutations were located in the channel domain of the CNGB1 protein.

Only one patient was found to harbor other IRD-related variants. P10 was heterozygous for a class IV variant in the COL9A2 gene (NM_001852.3:c. 150 + 1G > C p.?).

Segregation analysis and pedigrees are represented in Supplemental Figure S1.

### 3.2. Ocular Phenotype

All patients reported night blindness as the first visual symptom. As observed in Table 1, patient 6D was the only one referring an age of onset (AO) in mid-adulthood (30–50 years), with the remaining 14 subjects reporting an AO before 20 years, and 11/15 referring childhood onset (<10 years old), contrasting to the fact that diagnosis was only established after 30 years old in 9/15 (mean 40.67 ± 21.86, range 11–84). A relatively preserved VA was observed during the follow-up: mean VA varied from 74.33 (range 65–85) and 75.80 (range 55–85) ETDRS letters in the right and left eye, respectively, at the first visit; to 61.73 (range 0–85) and 67.27 (range 0–85) in the last visit (mean time follow-up 192.47 ± 154.60, range 24–516 months); these variations were not statistically significant (*p* = 0.74 and *p* = 0.51 for the right and left eye, respectively).

On CFP, all subjects except one (7E) showed bone spicule pigmentation, with 10/15 showing a distribution of this finding in the extreme and/or mid periphery and only 4 patients presenting bone spicule in the posterior pole beyond vascular arcades. All subjects presented signs of widespread retinal degeneration with patches of outer retinal atrophy, and 9/15 showed optic disc pallor while 13/15 presented narrowed retinal vessels.

Regarding FAF imaging, a central hyperautofluorescence ring limiting posteriorly an area of heterogeneous hypoautofluorescence was observed in 9/15 patients, with the pattern of a small ring (<1 disc diameter) being the most common (5/9 patients).

**Figure 2:**
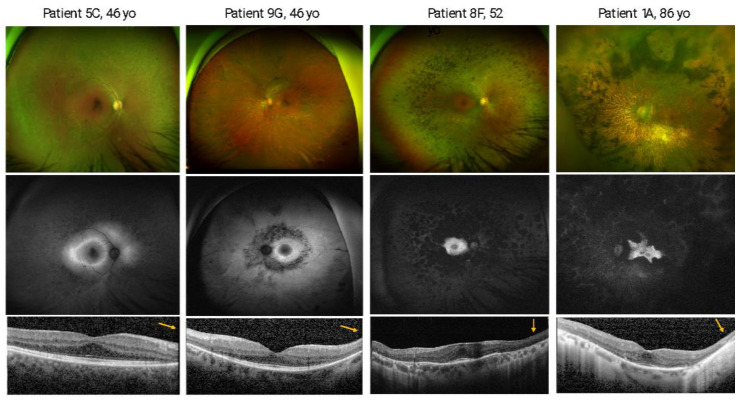
Retinal phenotype spectrum of the study population. For patients 5, 9, 8, and 1, ultra-widefield (UWF) color fundus photography (top), UWF-fundus autofluorescence (middle) and spectral domain optical coherence tomography (SD-OCT, bottom) images are depicted. Only 1 eye is shown in all cases due to high inter-eye symmetry. The cases shown from left to right reflect increasing disease severity in the different imaging modalities. The orange arrows indicate the SD-OCT b-scan direction.

On SD-OCT, 90% of the eyes presented subfoveal sparing of the ellipsoid zone line and retinal pigment epithelium, with only 3 patients (6D, 12I, and 13I) showing loss of these components in one of the eyes (Table 1). Epiretinal membrane (ERM) was observed in 10% of the eyes, whereas cystoid macular edema (CMO) was present in 8/30 eyes (Table 1). Figure 2 shows examples of the retinal phenotype of the study population.

### 3.3. Olfactory Phenotype

None of the patients had chronic rhinosinusitis or other known pathology associated with olfactory loss. Only two patients denied olfactory changes on subjective evaluation (VAS score 10), while ten patients reported moderate olfactory loss (VAS score between 4 and 6), and three patients stated they had no sense of smell (VAS score 0). Eight of the patients reported lifelong olfactory dysfunction, and one of those stated it had gotten worse since being infected by COVID-19. Four of the patients realized they had a subnormal sense of smell before they noticed vision loss. One patient reported not being able to detect a gas leak at home on one occasion. One of the normosmia patients had already been tested for olfactory threshold in 2006 for reporting olfactory dysfunction—she has stopped smoking since then, and her threshold actually increased in this assessment.

Regarding the odor threshold encountered with the CCCRC test, six patients were identified as having anosmia, three patients had severe hyposmia, one patient with moderate hyposmia, three patients presented mild hyposmia, and two patients had normosmia (Table 1). In the odor identification test, when estimated according to the most recent normative data [28], the SnSt identification test revealed that all but one of the Portuguese and Turkish patients tested below the cutoff for hyposmia, while all three of the French patients were found to have normosmia (Table 1).

## 4. Discussion

Central nervous system involvement is the most frequent non-ocular manifestation in syndromic forms of IRDs [29]. Despite being rare, the association between retinal degeneration and olfactory dysfunction has been reported in a few cases of Bardet-Biedl syndrome, Leber congenital amaurosis, and Refsum disease [30]. Recently, olfactory dysfunction in association with retinal degeneration has been described in *CNGB1*-related disease and later coined RP-olfactory dysfunction syndrome [6,14,15]. In this multicenter, multiethnic study, we expand the mutational spectrum of *CNGB1*-related disease by reporting two novel disease-causing variants. Additionally, we thoroughly characterized the retinal and olfactory phenotypes of 15 *CNGB1*-related retinal degeneration patients from ten different families and found that only 4 patients had a preserved sense of smell.

As expected from previous studies, olfactory self-assessment is not reliable as patients tend to undervalue their olfactory dysfunction [31,32,33]. That is why testing for olfactory function should include a psychophysical assessment with validated tests of odor threshold and at least one of odor identification or discrimination [25,34]. In our cohort, psychophysical testing confirmed olfactory dysfunction in association with several *CNGB1* mutations.

The fact that eight of our patients reported lifelong olfactory dysfunction while only presenting RP-related symptoms in adolescence/young adulthood raises the need to ask patients about their olfactory function and eventually test it in RP patients with late presentation of visual symptoms [14].

In our study, we identified a total of seven disease-causing variants in the *CNGB1* gene: two splice-site, two nonsense, one frameshift, and two missense; two of the mutations found (c.2565_2566del and c.2285G > T) are herein reported for the first time. The splice site c.1958-1G > A, p.? variant seems to be very prevalent in the Portuguese population, as all but one family harbored this mutation. When in homozygosity (4 families), all cases presented hyposmia or functional anosmia. However, when present in heterozygosity, the results varied. In the family presenting this variant in compound heterozygosity with the novel variant c.2565_2566del, p.(Phe856*), both patients had hyposmia; contrarily, in the only case where the variant was present in compound heterozygosity with the novel variant c.2285G > T, p.(Arg762Leu), the patient was normosmic. Interestingly, the c.2285G > T variant is one of the two missense mutations in our cohort, thus having a presumed milder effect, which may explain the lack of olfactory dysfunction in this patient. Furthermore, even though located in the channel domain, the c.2285G > T variant affects the N-terminal region of CNGB1 (amino acids 677–764), which plays an important role in the subunit interaction with a C-terminal region of CNGA1 [35], only present in rod cells, and thus probably not affecting the OSNs. In the French family, the previously described [13] c.2978G > T, p.Gly993Val variant (also a missense mutation) was observed in homozygosity, and all subjects presented normosmia in the odor identification test. We believe these missense variants may have a milder effect on olfactory function, even though they are both located in the channel domain and thus expected to cause olfactory dysfunction according to previous theories [6,13,15]. Since none of our families harbored variants in the GARP domain (not expressed in the OSNs), we could not assess the olfactory phenotype of mutations affecting this protein domain. However, previous studies [6,14] reported hyposmia in patients with homozygous mutations located in the GARP domain, suggesting that the olfactory function consequences cannot be solely explained by the affected protein domain [6,14,15]. The modifying role that has been attributed to CNGB1 in the olfactory CNG channels could eventually explain some residual function even when integrating only CNGA2 and CNGA4 subunits [6].

The Turkish family harbored the mutation c.2492 + 2T > G, which has been suggested to have a prominent role in olfactory dysfunction, even in patients who were carriers of a single mutation and hence did not show retinal involvement [14]. Since our study only included patients with retinal disease, we could not confirm this hypothesis, but we did find that both patients harboring that variant (14J in homozygosity and 15J in compound heterozygosity) had functional anosmia. In patient 14J, the retinal phenotype was mild, supporting the dominant effect of that mutation on the olfactory function.

Regarding the ocular phenotype, all patients reported nyctalopia as their first visual symptom and 73.33% referred an early onset (i.e., during childhood). Nevertheless, a diagnosis of RP was only established after 30 years of age in 60% of the individuals, and a relatively preserved VA was found until the fifth decade for the majority of patients. These results are in accordance with the literature, which refers to *CNGB1*-related RP as an early onset but relatively benign RP, allowing a moderately preserved visual function until the late stages of the disease [6,13]. An early onset of nyctalopia in the absence of marked retinal degeneration is explained by CNGB1 subunits’ crucial role in rod photoreceptor activity.

Both intra-familial and inter-familial phenotypic variability was observed in our cohort, as exemplified by individuals 11I and 14l, who presented a later onset (during adolescence), compared to an earlier onset in the remaining relatives with the same mutations. Moreover, the retinal phenotype seemed not to differ according to the variant’s functional effect, since less severe phenotypes were not observed in families carrying missense mutations. Nevertheless, the number of reported cases is still too small to draw conclusions regarding the influence of mutation type on disease severity [6,18].

Regarding multimodal retinal imaging, a hyperAF ring was observed in 60% of patients, a frequency slightly inferior to what has been previously reported in CNGB1-related RP [13]. In SD-OCT, the prevalence of CMO (26.67%) in our cohort was superior to that previously reported in *CNGB1*-related RP [13,18] but in line with the frequency of CMO in syndromic and non-syndromic RP [36].

It is important to note, however, that the study population (*n* = 15) is limited and hence not enough to define the effect of identified mutations in RP and olfactory dysfunction. Therefore, studies with larger multiethnic samples and meta-analyses incorporating smaller studies like this one are needed to confirm the effect of the identified mutations. Another limitation of our work lies in the fact that genetic testing focuses exclusively on IRD-associated genes. Thus, we cannot exclude the presence of further genetic variants in olfactory-specific genes that could affect the observed olfactory phenotype.

## 5. Conclusions

In conclusion, our study supports previous reports of an autosomal recessive *RP-olfactory dysfunction syndrome* in association with certain disease-causing variants in the *CNGB1* gene and expands the mutational spectrum of *CNGB1*-related disease by reporting two novel *CNGB1* variants. Patients harboring missense variants (in homo- or compound heterozygosity) did not present olfactory dysfunction, highlighting the role of variant characterization in predicting olfactory involvement. Further studies are needed to verify this hypothesis, aiming to establish definite genotype-phenotype correlations.

## Figures and Tables

**Table 1:** Demographic, clinical, and genetic data of the study population.

Patient (Gender; Age)	Family	Nationality	*CNGB1* Variants (NM_001297.4) (ACMG Classification)		Consanguinity	Family History	Age of Onset	Age at Diagnosis (Years)	VA—First Visit (ETDRS Letters)		VA—Last Visit (ETDRS Letters)		RPE/EZ Foveal Sparing on SD-OCT (Last Visit)		Olfactory Phenotype	
			V1	V2					OD	OS	OD	OS	OD	OS	Odor Threshold—CCCRC Test Score (Classification)	Odor Identification—SnSt 16 Pens Score (Classification) ^a^
1 (F; 86)	A	PT	c.1958-1G > A p.? (V)	c.1958-1G > A p.? (V)	-	+	Childhood	84	66	69	66	69	+	+	0 (Anosmia)	1 (Functional anosmia)
2 (M;77)	A					+	Childhood	74	65	65	67	62	+; VMT	+;VMT	0 (Anosmia)	2 (Functional anosmia)
3 (M;55)	B	PT	c.3150del p.(Phe1051Leufs*12) (V)	c.3150del p.(Phe1051Leufs*12) (V)	-	-	Childhood	52	81	81	85	75	+	+	0 (Anosmia)	5 (Functional anosmia)
4 (M;42)	C	PT	c.1958-1G > A p.? (V)	c.2565_2566del p.(Phe856*) (IV)	-	+	Childhood	11	85	85	85	85	+; CMO	+; CMO	2 (Severe hyposmia)	8 (Hyposmia)
5 (F;46)	C					+	Childhood	15	85	85	85	80	+	+	5 (Mild hyposmia)	8 (Hyposmia)
6 (M;71)	D	PT	c.1958-1G > A p.? (V)	c.2285G > T p.(Arg762Leu) (IV)	-	+	Mid adulthood	49	75	55	70	0	+;VMT	-;VMT	5 (Mild hyposmia)	12 (Normosmia)
7 (F;54)	E	PT	c.1958-1G > A p.? (V)	c.1958-1G > A p.? (V)	+	-	Childhood	48	80	80	77	76	+	+	1 (Anosmia)	8 (Functional anosmia)
8 (F;52)	F	PT	c.1958-1G > A p.? (V)	c.1958-1G > A p.? (V)	-	+	Adolescence	44	77	79	65	65	+;ERM	+;ERM	2 (Severe hyposmia)	11 (Hyposmia)
9 (M;46)	G	PT	c.1958-1G > A p.? (V)	c.1958-1G > A p.? (V)	+	+	Childhood	16	85	85	0	80	Impossible to evaluate (dense cataract)	+	5 (Mild hyposmia)	10 (Hyposmia)
10 (F;77)	H	PT	c.1958-1G > A p.? (V)	c.1958-1G > A p.? (V)	-	+	Childhood	67	70	60	55	44	+;CMO	+; CMO	0 (Anosmia)	2 (Functional anosmia)
11 (M;63)	I	FR	c.2978G > T, p.Gly993Val (IV)	c.2978G > T, p.Gly993Val (IV)	-	+	Adolescence	20	50	75	50	75	+	+; LH	6 (Normosmia)	14 (Normosmia)
12 (M;60)	I	FR				+	Childhood	38	65	80	35	75	-; LH	+; LH	6 (Normosmia)	14 (Normosmia)
13 (F;55)	I	FR				+	Childhood	30	75	85	65	75	-; CMO	+; CMO, ERM	4 (Moderate hyposmia)	13 (Normosmia)
14 (M;49)	J	TR	c.2492 + 2T > G, p.? (V)	c.2492 + 2T > G, p.? (V)	+	+	Adolescence	44	85	85	75	80	+	+	1 (Anosmia)	4 (Functional anosmia)
15 (F;24)	J	TR	c.1917G > A, p.(Trp639*) (V)	c.2492 + 2T > G, p.? (V)		+	Childhood	18	75	80	75	80	+; CMO	+; CMO	2 (Severe hyposmia)	8 (Functional anosmia)

F—female; M—male; PT—Portugal; FR—France; TR—Turkey; V1—first variant; V2—second variant; VA—visual acuity; EPR/EZ—retinal pigment epithelium/ellipsoid zone; SD-OCT—spectral domain optical coherence tomography; VMT—vitreous macular traction; CMO—cystoid macular oedema; ERM—epiretinal membrane; LH—lamellar hole; CCCRC—Connecticut Chemosensory Clinical Research Center; SnSt—Sniffin’ Sticks^®^. ^a^ According to the updated Sniffin’ Sticks normative data (Oleszkiewicz A et al. Updated Sniffin’ Sticks normative data based on an extended sample of 9139 subjects. Eur Arch Oto Rhino Laryngol. 2019; 276: 719–728.).

## Data Availability

Data used for analysis are anonymously available upon request.

## Supplementary Materials

The following supporting information can be downloaded at: https://www.mdpi.com/article/10.3390/genes14040830/s1, Figure S1: Pedigrees of the families included in the study; Table S1. Genes included in the NGS panels used.
