# Supplementary material for: Mutational Spectrum, Ocular and Olfactory Phenotypes of CNGB1-Related RP-Olfactory Dysfunction Syndrome in a Multiethnic Cohort
Source: Genes (Basel). 2023 Mar 30;14(4):830. doi: 10.3390/genes14040830 (PMC10137467; doi:10.3390/genes14040830)
Supplement: Supplementary file 1 [file genes-14-00830-s001.zip › genes-2215515-supplementary.pdf]

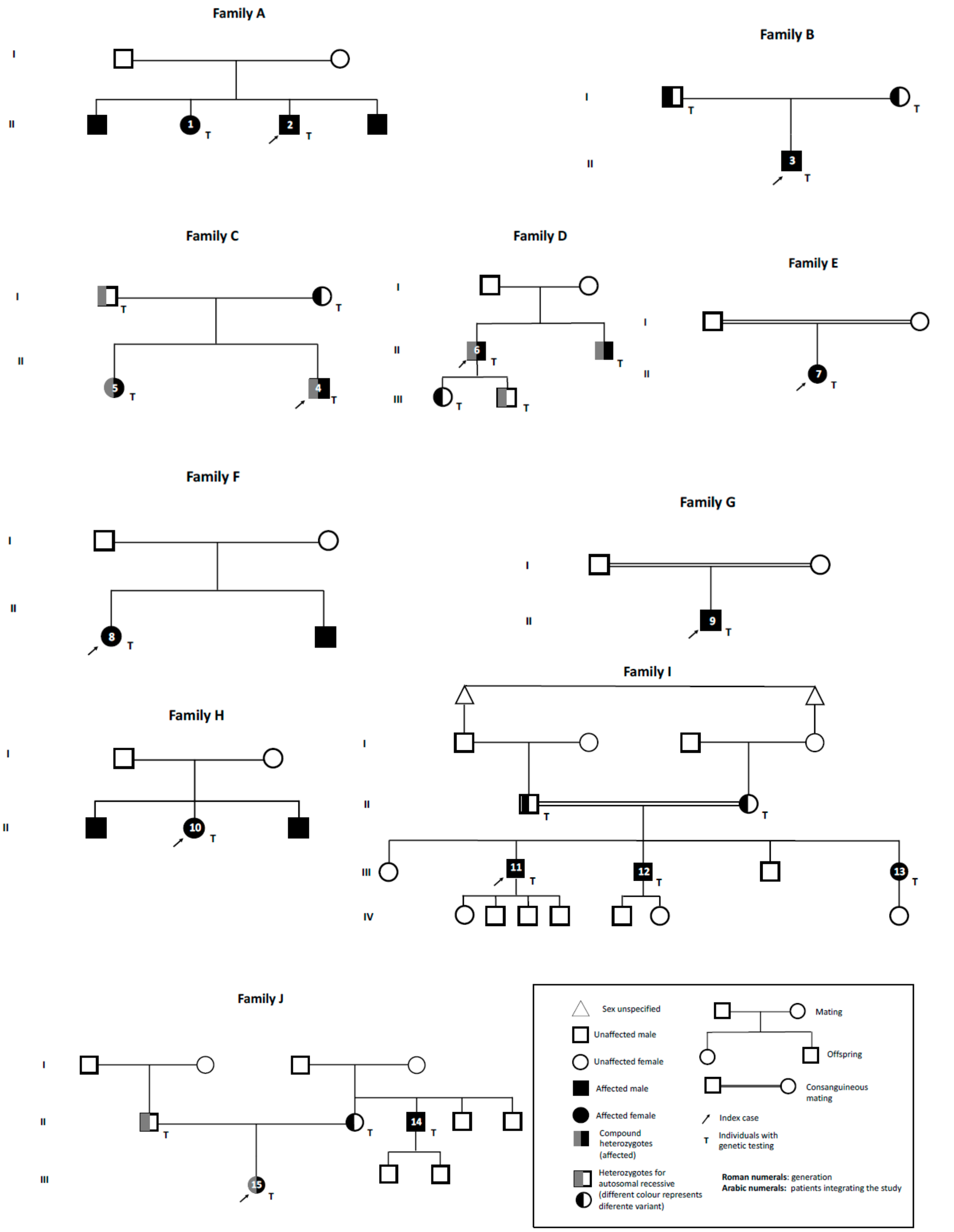

Supplemental Figure S1. Pedigrees of the families included in the study

WES-based NGS panel (302 IRD-associated genes)	IRD NGS panel (238 IRD-associated genes)
ABCA4, ABHD12, ACBD5, ACO2, ADAM9, ADAMTS18, ADGRA3, ADGRV1, AGBL5, AHI1, AIPL1, ALMS1, AMN, ARHGEF18, ARL13B, ARL2BP, ARL6, ARMS2, ATF6, ATOH7, ATP13A2, B3GLCT, BBIP1, BBS1, BBS10, BBS12, BBS2, BBS4, BBS5, BBS7, BBS9, BCOR, BEST1, BMP4, C1QTNF5,	*ABCA4, ABHD12, ADAM9, ADAMTS18, ADIPOR1, AGBL5, AHI1, AHR, AIPL1, ALMS1, ARHGEF18, ARL2, ARL2BP, ARL3, ARL6, ARSG, ASRGL1, ATF6, ATXN7, BBS1, BBS2, BBS10, BBS12, BEST1, C12orf65, C1QTNF5, C21orf2, C2orf71, C8orf37, CA4, CABP4, CACNA1F, CACNA2D4, CAPN5, CC2D2A, CCT2, CDH23, CDH3, CDHR1, CEP164/NPHP15, CEP250,*

C2, C21orf2, C2orf71, C3, C5orf42, C8orf37, CA4, CABP4, CACNA1F, CACNA2D4, CAPN5, CC2D2A, CDH23, CDH3, CDHR1, CEP164, CEP290, CEP41, CEP78, CERKL, CFB, CFH, CHM, CIB2, CLN3, CLN5, CLN6, CLN8, CLRN1, CNGA1, CNGA3, CNGB1, CNGB3, CNNM4, COL11A1, COL11A2, COL18A1, COL2A1, COL4A1, COL9A1, COL9A2, CRB1, CRX, CSPP1, CTNNB1, CTSD, CUBN, CWC27, CYP1B1, CYP27A1, CYP2R1, CYP4V2, DHDDS, DHX38, DTHD1, EFEMP1, ELOVL4, EMC1, ERCC6, ERCC8, EYS, FAM161A, FAM57B, FAM71A, FBLN5, FLVCR1, FOXC1, FOXE3, FOXI2, FRAS1, FREM1, FREM2, FSCN2, FZD4, GDF6, GNAT1, GNAT2, GNPTG, GP1BA, GPR143, GPR179, GRIP1, GRK1, GRM6, GRN, GUCA1A, GUCA1B, GUCY2D, HARS, HCCS, HGSNAT, HK1, HMCN1, HMX1, HTRA1, IDH3B, IFT140, IMPDH1, IMPG1, IMPG2, INPP5E, INVS, IQCB1, IRX5, ITM2B, KCNJ13, KCNV2, KCTD7, KIAA1549, KIF11, KIF7, KIZ, KLHL7, LCA5, LRAT, LRIT3, LRMDA, LRP2, LRP5, LZTFL1, MAK, MERTK, MFN2, MFRP, MFSD8, MKKS, MKS1, MTTP, MVK, MYO7A, MYOC, NAALADL1, NDP, NEK2, NMNAT1, NPHP1, NPHP3, NPHP4, NR2E3, NR2F1, NRL, NUMB, NYX, OAT, OCA2, OFD1, OPA1, OPA3, OPN1LW, OPN1MW, OTX2, P3H2, PANK2, PAX6, PCDH15, PCYT1A, PDAP1, PDE6A, PDE6B, PDE6C, PDE6G, PDE6H, PDZD7, PEX1, PEX2, PEX7, PHYH, PITPNM3, PITX2, PITX3, PLA2G5, PLD4, POC1B, PPT1, PRCD, PROM1, PRPF3, PRPF31, PRPF4, PRPF6, PRPF8, PRPH2, PRPS1, PRTFDC1, RAB28, RAX2, RB1, RBP3, RBP4, RCBTB1, RD3, RDH12, RDH5, RGR, RGS9, RGS9BP, RHO, RIMS1, RLBP1, ROM1, RP1, RP1L1, RP2, RP9, RPE65, RPGR, RPGRIP1, RPGRIP1L, RS1, SAG, SCAPER, SDCCAG8, SEMA4A, SLC24A1, SLC24A5, SLC37A3, SLC38A8, SLC45A2, SLC7A14, SMOC1, SNRNP200, SOX2, SPATA7, SPG7, SRD5A3, STRA6, TCTN1, TCTN2, TCTN3, TEAD1, TEX28, TIMM8A, TIMP3, TMEM126A, TMEM216, TMEM237, TMEM67, TOPORS, TPP1, TRIM32, TRPM1, TSPAN12, TTC21B, TTC8, TTLL5, TUB, TULP1, TYR, TYRP1, UBAP1L, UNC119, USH1C, USH1G, USH2A, VAX1, VCAN, VPS13B, VSX2, WASF3, WDPCP, WDR19, WFS1, WHRN, WT1, ZNF408, ZNF423, ZNF513.	*CEP290/NPHP6, CEP78, CERKL, CFH, CHM, CLCC1, CLN3, CLN5, CLN6, CLN8, CLRN1, CLUAP1, CNGA1, CNGA3, CNGB1, CNGB3, CNNM4, COL18A1, COL4A5, CRB1, CRB2, CRX, CSPP1, CTNNA1, CYP4V2, DHDDS, DHX38, DRAM2, DTHD1, EFEMP1, ELOVL4, EMC1, EYS, FAM161A, FLVCR1, FRMD7, FSCN2, FZD4, GDF6, GNAT1, GNAT2, GNB3, GPR125/ADGRA3, GPR179, GRK1, GRM6, GUCA1A, GUCA1B, GUCY2D, HARS, HGSNAT, HK1, HMCN1, IDH3A, IDH3B, IFT140, IFT172, IFT81, IMPDH1, IMPG1, IMPG2, INPP5E, INVS/NPHP2, IQCB1/NPHP5, ITM2B, JAG1, KCNJ13, KCNV2, KIAA1549, KIF11, KLHL7, LAMA1, LCA5, LRAT, LRIT3, LRP5, MAK, MAPKAPK3, MERTK, MFRP, MFSD8, MKKS, MMACHC, MTTP, MVK, NDP, NEK2, NEUROD1, NMNAT1, NPHP1, NPHP3, NPHP4, NR2E3, NRL, NYX, OAT, OFD1, OPN1LW, OPN1MW, OPN1SW, OTX2, PDE6A, PDE6B, PDE6C, PDE6G, PDE6H, PDZD7, PEX1, PEX6, PEX7, PEX26, PGK1, PHYH, PITPNM3, PLA2G5, PLK1S1/KIZ, PNPLA6, POC1B, POC5, POMGNT1, PRCD, PRDM13, PROM1, PRPF3, PRPF4, PRPF6, PRPF8, PRPF31, PRPH2, PYGM, RAB28, RAX2, RBP3, RBP4, RCBTB1, RD3, RDH11, RDH12, RDH5, REEP6, RGR, RGS9, RGS9BP, RHO, RIMS1, RIMS2, RLBP1, ROM1, RP1, RP1L1, RP2, RP9, RPE65, RPGR, RPGR, ORF15, RPGRIP1, RS1, SAG, SAMD11, NPHP10/SDCCAG8, SEMA4A, SLC24A1, SLC7A14, SNRNP200, SPATA7, SPP2, TIMP3, TMEM216, TOPORS, TRNT1, TRPM1, TSPAN12, TTC8, TTLL5, TUB, TUBGCP4, TUBGCP6, TULP1, UNC119, USH2A, VCAN, VPS13B, WDR19, ZNF408, ZNF423, ZNF513*

Supplemental Table S1. Genes included in the NGS panels used.
